# The role of phototherapy in pediatric dermatology^؆^

**DOI:** 10.1016/j.abd.2025.501252

**Published:** 2025-12-26

**Authors:** Eine Benavides, Dan Hartmann, Catalina Retamal, Fernando Valenzuela

**Affiliations:** a Hospital Universitario del Valle “Evaristo García”, Universidad del Valle, Cali, Colombia; b Department of Dermatology, Faculty of Medicine, Universidad de Chile, Santiago, Chile; c Department of Dermatology, Faculty of Medicine, Universidad de los Andes, Santiago, Chile

**Keywords:** Phototherapy, Psoriasis, PUVA therapy, Ultraviolet therapy, Vitiligo

## Abstract

**Background:**

Phototherapy is one of the widely used therapeutic options in dermatology, and it has proven effective for many dermatological conditions. It includes various modalities such as heliotherapy, broad-band UVB, narrow-band UVB, excimer laser, UVA1, UVA with Psoralens (PUVA), among others. The mechanisms behind phototherapy's efficacy include proapoptotic, immunomodulatory, propigmenting, antifibrotic, and antipruritic effects. In this context, the effectiveness of this modality has been demonstrated in pediatric patients with various conditions; however, no consensus has yet been established regarding its use in this population.

**Methods:**

A comprehensive literature review was conducted to identify the most recent studies and advancements in the use of phototherapy in the pediatric population.

**Results:**

Phototherapy is a safe and effective therapeutic modality that can be used in multiple conditions, such as psoriasis, vitiligo, atopic dermatitis, mycosis fungoides, pityriasis lichenoides, and actinic prurigo, among others. The therapeutic outcomes depend on the condition being treated, the type of phototherapy used, and the appropriate selection of patients.

**Conclusions:**

The phototherapy with NB-UVB is the most commonly use and preferred modality due to its efficacy and lower risk associated. Careful monitoring is recommended to assess long-term safety and optimize pediatric treatment protocols.

## Introduction

Phototherapy is a treatment that involves delivering Ultraviolet (UV) radiation to patients to treat various dermatologic conditions^1^ and has been highly effective in the management of epidermal and deep dermal diseases.^2^ While it is an effective and safe treatment option for many skin disorders in adults, its use in children has been more restricted due to concerns about the potential long-term carcinogenic risks associated with UV exposure.^3^ Nonetheless, there is substantial evidence supporting its use in pediatric dermatology for conditions such as vitiligo, Atopic Dermatitis (AD), psoriasis, alopecia areata, pityriasis lichenoides, mycosis fungoides, scleroderma, among others.^4^

Phototherapy has been practiced since ancient times, with records of heliotherapy and exposure to sunlight in ancient Egypt, China, and Hindu culture. The authors have the Ebers Papyrus (1550 BCE), which contains the treatment of vitiligo with Psoralen corylifolia and Ammi majus extract, and the after exposure of the person to sunlight. And many more records. In the 19th century, Nils Ryberg Finsen (1860‒1904) started using electric carbon arc torch in the treatment of patients with lupus vulgaris, a chronic and progressive form of cutaneous tuberculosis caused by the bacterium Mycobacterium tuberculosis, becoming the father of modern phototherapy, and subsequently, the use of artificial light in phototherapy took place in the treatment of different skin diseases, till William Henry Goeckerman (1884‒1954) began using UVB light to treat psoriasis. Since that, the field of phototherapy has been constantly growing and expanding to different areas of medicine such as neonatology, infectology, psychiatry, ophthalmology, rheumatology, and oncology.^5^

Photodermatology is a crucial part of the dermatological practice and requires appropriate knowledge and expertise for the correct implementation of this treatment.^2^ The mechanism by which phototherapy works varies depending on the specific condition being treated and the modality employed. However, it is generally understood that UV light helps to modulate the immune response that underlies many inflammatory and autoimmune skin diseases, induces apoptosis, and modifies the cytokine environment.^6^, ^7^

Phototherapy mechanisms of action that can occur simultaneously, among them are: proapoptotic effects (AD, psoriasis, T-cell lymphomas), immunomodulatory effects (AD, psoriasis, T-cell lymphomas), propigmenting effects (vitiligo), antifibrotic effects (scleroderma, GVHD), antipruritic effects (various dermatoses where pruritus is a predominant symptom), and, probiotic and prebiotic effects (AD, psoriasis).^2^ Treatment options include heliotherapy (sunlight exposure), UVA1 (340–400 nm), UVA (320–400 nm), either alone or in combination with Psoralen (PUVA), broadband UVB (BB-UVB: 280–320 nm), narrowband UVB (NB-UVB: 311–313 nm), the excimer laser (UVB: 308 nm), as well as combination therapies that involve both UVA and UVB.^2^, ^4^, ^8^

Despite the existing body of evidence, there is a notable lack of randomized controlled trials on pediatric phototherapy, with most data coming from retrospective studies. As a result, treatment protocols and dosing regimens are often based on clinical experience rather than standardized guidelines. Additionally, special considerations must be considered when administering phototherapy to children, including the safe delivery of treatments, appropriate scheduling, and addressing long-term safety concerns.^4^, ^9^, ^10^ This review aims to highlight the primary indications for phototherapy in children and explore the critical considerations involved in administering UV treatments within the pediatric population.

## Material and methods

During November 2024 and February 2025, the authors conducted a narrative review of the literature by entering the terms “phototherapy”, “pediatric dermatology”, “NB-UVB”, “PUVA”, “UVA1”, “excimer light”, “psoriasis phototherapy”, “vitiligo phototherapy” and “atopic dermatitis phototherapy” into PubMed and Google Scholar. The search was limited to articles in English and Spanish. All three authors participated in the search and subsequently selected the articles based on their relevance.

### Indications

Principal indications, type and modality of phototherapy are summarized in Table 1.

**Table 1:** Principal indications, type and modality of phototherapy in pediatric dermatology.

Indication	Modality used	Clinical comment
Psoriasis	UVB (nb-UVB, bb-UVB)	High efficacy, well-tolerated, prolonged remission in some cases
	Excimer laser	
	PUVA.	
Vitiligo	nb-UVB	NB-UVB is the most effective treatment, providing superior facial and neck repigmentation; its effects are enhanced when combined with topical steroids or tacrolimus.
	Excimer laser	
	PUVA	
	UVA1+UVB	
Atopic dermatitis	NB-UVB	Significant improvement; well-tolerated
	UVA	
Cutaneous T-cell lymphoma	nb-UVB	High remission rates; PUVA with longer remission duration
	PUVA	
Actinic prurigo	UVB (nb-UVB, bb-UVB)	Temporary symptom relief: seasonal prophylactic use recommended
	PUVA	
Pityriasis lichenoides	nb-UVB.	High remission rate
Alopecia areata	nb-UVB	Poor efficacy; excimer partially effective; not recommended as standard
	Excimer laser	
	PUVA	
Morphea / Localized scleroderma	nb-UVB	Effective in superficial, non-progressive forms; preferred in children >12-years
	UVA1	
	PUVA	
Mastocytosis	nb-UVB	Reduced pruritus and serum tryptase
	PUVA	
Langerhans Cell Histiocytosis	nb-UVB	Complete resolution of cutaneous lesions with no adverse effects
Graft-versus-host disease	nb-UVB	High response rates; NB-UVB with better safety profile
	PUVA	
	UVA1	
Prurigo nodularis	nb-UVB	Significant improvement; incomplete follow-up
Other conditions (urticaria pigmentosa, etc.)	nb-UVB	Used as photoprophylaxis and therapy for pruritic dermatoses
	PUVA	

### Psoriasis

**Fig. 1:**
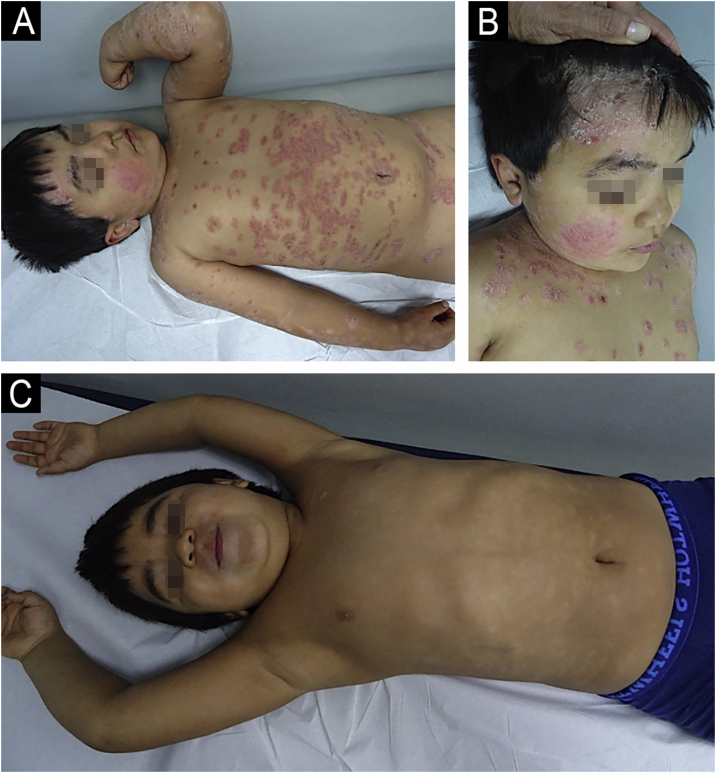
(A) Six-year-old male patient diagnosed with psoriasis, presenting with intensely pruritic, erythematous, and scaly plaques on the face, scalp, and both anterior and posterior trunk. (B) Same patient after 22 biweekly sessions of nb-UVB phototherapy, showing significant clinical improvement with marked reduction in erythema, scaling, and pruritus.

Psoriasis corresponds to a chronic multifactorial inflammatory skin disease, caused by a dysregulation of the immune system characterized by an activation of T-cells and proliferation of keratinocytes, affecting primarily skin, nails, and joints, with the presence of erythematous-desquamative plaques in the face, body, and extremities.^11^ It has a prevalence of 2%–3% of the global population, and it represents 4% of all dermatoses in patients younger than 16-years-old.^12^ The treatment is based on a step-wise approach, with the use of topical treatment in mild cases (topical corticosteroids, calcineurin inhibitors, and vitamin D derivatives) and systemic therapy in moderate and severe cases (systemic immunomodulators, biological agents, phototherapy, or photochemotherapy).^13^ The evidence supporting the use of phototherapy in psoriasis primarily comes from retrospective reviews.^14^ The American Academy of Dermatology (AAD) recommends phototherapy as a second-line treatment for children with psoriasis who do not respond to initial topical therapies or in cases of extensive disease.^15^, ^16^ NB-UVB is the preferred modality due to its proven efficacy, safety, and ease of administration in pediatric patients with psoriasis (Fig. 1A‒B). In one of the largest studies to date, 88 children with psoriasis were treated using NB-UVB, with 92% of patients experiencing more than 75% improvement in their condition, with full clearance achieved in 51%.^17^ A prospective study of 20 pediatric patients with recurrent guttate or plaque psoriasis showed an improvement of a minimum of 60% after 12-weeks of treatment, with remission rates of 90%.^18^ Other treatment modalities include BB-UVB, excimer laser, and PUVA. In a study involving 30 patients with psoriasis (mean age 11 ± 3.6 years) treated with BB-UVB (mean number of treatments: 28.8 ± 13.3), 93.3% of subjects experienced more than 75% improvement. Another study that compared the safety and efficacy of the excimer laser in adults versus children evidenced efficacy and safety in both groups, with 12.5 sessions in children compared with the 9.7 sessions needed in adults.^19^ Additionally, a small cohort of seven children with plaque or guttate psoriasis was treated with PUVA therapy, resulting in more than 75% clinical improvement in 83% of patients after an average of 28 treatment sessions.^20^ To assess long-term efficacy, a retrospective study involving 75 pediatric patients aged 3 to 17 years evaluated the durability of phototherapy. After 12-months, 52% of the 21 patients with psoriasis remained free of clinical symptoms.^1^ Phototherapy can be used alone, or combined with other treatments such as emollients (e.g. mineral oil), topical corticosteroids, topical calcineurin inhibitors, coal tar (Goeckerman treatment), retinoids, among others.^13^, ^14^ Thus, both NB-UVB and PUVA can be safely and effectively administered to pediatric patients using the same treatment protocols as those applied in adults.^21^

### Vitiligo

**Fig. 2:**
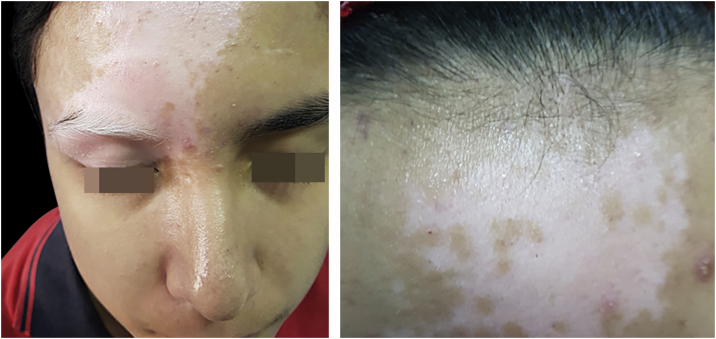
(A) Fourteen-year-old male patient diagnosed with vitiligo, presenting with an achromic patch involving the glabellar region and extending to the right upper eyelid, associated with poliosis of the right upper eyelashes. (B) Same patient after 12 biweekly sessions of 308-nm excimer light phototherapy with good responses and signs of repigmentation.

Vitiligo is an acquired chronic autoimmune disease that is characterized by the presence of white macules and patches, secondary to a progressive loss of melanocytes and alteration in their functions.^22^ It has a prevalence of 0.1%–4% of the global population, and approximately 50% develop by 20-years.^23^ Topical therapies are the first-line treatment for children with vitiligo, but phototherapy may be considered if these treatments fail or in cases of extensive or rapidly progressive disease.^24^ Several phototherapy modalities have been used in pediatric vitiligo, including NB-UVB, PUVA, combined UVA1 and UVB, and the 308-nm excimer laser (Fig. 2). Historically, PUVA was frequently used to treat vitiligo; however, it has largely been replaced by NB-UVB due to its superior repigmentation outcomes and the potential risks associated with PUVA in children, such as an increased risk of malignancy.^25^ The first study to explore NB-UVB use in childhood vitiligo was conducted by Njoo and colleagues, who found disease stabilization in about 80% of pediatric patients with generalized vitiligo treated with NB-UVB twice weekly. Over half of the patients experienced more than 75% repigmentation.^26^ Response to therapy was positively correlated with the location of the lesions, with better improvement seen in lesions on the face and neck, and less improvement in acral lesions. This is thought to be due to the lower density of hair follicles in acral areas, which reduces the ability of UV light to stimulate residual follicular melanocytes.^27^ For pediatric patients with treatment-resistant vitiligo, combination therapy using topical immunomodulatory agents alongside phototherapy may offer better results than phototherapy alone. One open-label study investigated the combination of NB-UVB and 0.03% tacrolimus ointment in children with symmetric vitiligo lesions. The study found a significant increase in repigmentation at 4- to 6-months with combination therapy compared to phototherapy alone, and patients required fewer phototherapy sessions and lower cumulative doses to achieve clinically visible responses.^28^ A retrospective review of 71 patients aged 5‒15 years with vitiligo, more than half of whom had generalized vitiligo and over a third had segmental vitiligo, found that patients with generalized vitiligo had a better response to treatment than those with segmental vitiligo. The study reported the highest response rates for NB-UVB phototherapy (74% response rate), followed by targeted phototherapy combining UVA1 and UVB (67% response). 308-nm excimer lamp phototherapy and PUVA had marginally lower response rates (54% and 53%, respectively). Treatment duration ranged from 3- to 40-months, with the number of treatments ranging from 20 to 209 sessions. Side effects were generally mild and included itching, scaling, erythema, pain, sunburn, blistering, and phototoxicity.^29^ In a retrospective chart review of 324 pediatric patients with vitiligo, 126 patients were treated with NB-UVB for a mean duration of 18-months, and Thirty-three patients (29%) experienced sunburn, including 10 (7.9%) who used home units; however, they were able to resume treatment after counseling.^30^ Factors associated with improved or resolved vitiligo included nonsegmental disease, fewer signs of active disease, involvement of the face, head, neck, and extremities, and more areas of involvement.^30^ Phototherapy should be considered for pediatric vitiligo unresponsive to topical agents with large body surface area involvement or advancing disease.^31^

### Atopic dermatitis

**Fig. 3:**
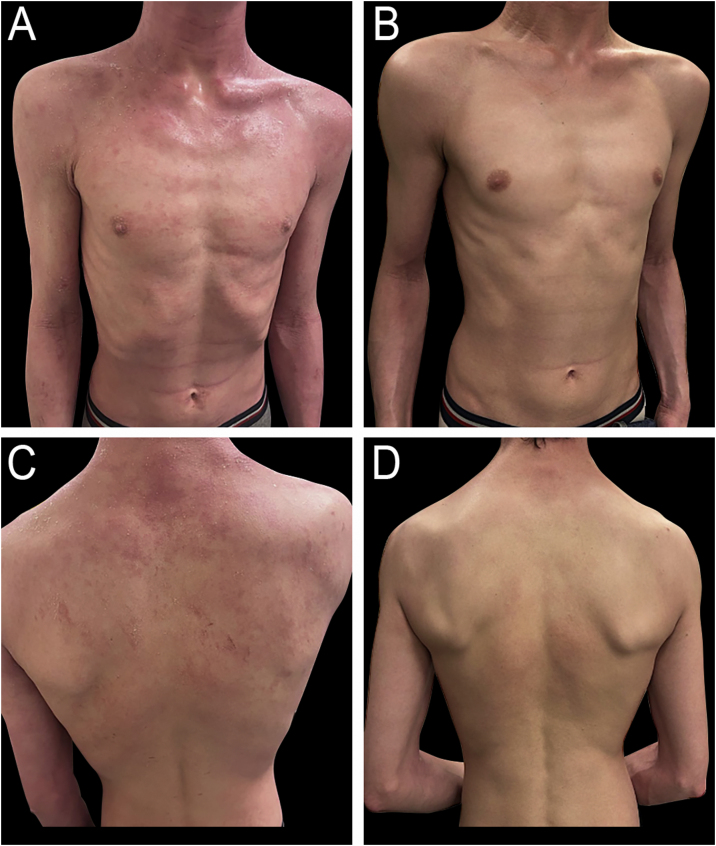
(A) Seventeen-year-old male patient diagnosed with atopic dermatitis presenting with pruritic, erythematous, and scaly plaques on the body and lower limbs. (B) Same patient after 18 biweekly sessions of nb-UVB phototherapy, showing significant clinical improvement.

Atopic Dermatitis (AD) is a chronic recurrent inflammatory disease that is characterized by the presence of pruritic eczematous patches in the skin. It is more frequent in childhood, affecting 10%–20% of the pediatric population.^32^ Topical therapy with emollients, steroids, and/or calcineurin inhibitors is the first-line treatment for children with AD. However, phototherapy is recommended as a second-line treatment for patients with moderate to severe AD who do not respond adequately to topical therapies.^4^, ^33^ The AAD lists both UVA and UVB as safe and effective treatments for childhood AD (Fig. 3A‒B), either as monotherapy or in combination with emollients and topical steroids.^34^ NB-UVB is the most studied light modality for pediatric AD, with numerous studies supporting its safety and efficacy. In one of the largest retrospective studies, Clayton and colleagues found that 40% of children with AD treated with NB-UVB achieved complete clearance or minimal residual disease, while 23% showed significant improvement. The median time to remission was approximately 3-months.^35^ Another study reported that more than half of their pediatric patients remained clear at one-year follow-up after completing NB-UVB therapy.^1^ A prospective cohort study of children ages 3‒16 years with moderate-to-severe AD showed a 61% reduction in mean SASSAD (Six Area, Six Sign Atopic Dermatitis) score in 29 patients who received phototherapy, compared to a 6% reduction in the 26 patients who deferred phototherapy. In addition, the mean surface area involvement at the end of treatment was 11% for the NB-UVB cohort, compared to 36% for the unexposed cohort.^36^ A retrospective study of 62 patients, aged 4 to 16, with moderate to severe AD showed that 56.9% of patients experienced treatment success, as indicated by improvements in Investigator Global Assessment and Eczema Area and Severity Index (EASI) scores. Common side effects included xerosis, pruritus, erythema, and pain. Other reasons for discontinuing NB-UVB therapy included difficulties with time commitment (9.3%), hyperactivity (2.3%), and claustrophobia (2.3%).^37^

### Cutaneous T-cell lymphoma

Mycosis Fungoides (MF) is the most common cutaneous T-cell lymphoma, but in the pediatric population, it is a rare disease, with a prevalence of 5%, but with an indolent course and with overall good prognosis.^38^ Phototherapy in pediatric patients with MF is considered a first-line treatment, with studies supporting the efficacy of both NB-UVB and PUVA. There are no established treatment guidelines for pediatric MF, as the progression of the disease in children is extremely rare and typically follows an indolent course. MF is often diagnosed in early stages (IA, IB, IIA), and phototherapy, with a response rate greater than 80%, is considered an effective treatment in these cases. However, since recurrences are frequently seen after therapy is discontinued, a maintenance regimen and long-term follow-up are essential.^39^ NB-UVB is recommended as first-line therapy for early-stage MF, particularly in younger patients with the hypopigmented form of the disease. In refractory cases, PUVA may be considered, but the potential long-term risks of PUVA should be carefully weighed against its benefits in managing this cutaneous malignancy on a case-by-case basis. While direct comparisons between NB-UVB and PUVA are lacking, some studies suggest that NB-UVB may be associated with more frequent recurrences than PUVA.^40^ In a study by Brazzelli and colleagues, the mean remission period for patients aged 15‒19 years was 11-months following NB-UVB treatment, compared to 30-months following PUVA. In those under 15-years-old, complete remission for a mean of 59-months was achieved with NBUVB alone.^32^ A prospective study of 23 patients with MF found that hypopigmented MF was the most common clinical presentation (52.2%), followed by classical MF (30.4%) and folliculotropic MF (17.4%). All patients were treated with topical corticosteroids and phototherapy. Complete remission was achieved in 59.1% of cases, and partial response was seen in 40.9%. Two patients remained asymptomatic for five years.^41^ NB-UVB has shown favorable outcomes in children with the hypopigmented variant of MF.^42^

### Actinic prurigo

Actinic Prurigo (AP) is a rare photodermatosis characterized by the presence of an intense pruritic papulonodular dermatitis that affects sun-exposed areas, typically the face, neck, and upper extremities, and appears after UV exposure.^43^ Its etiology is unclear, but 90% of the cases are associated with the HLA-DR4.^44^ It mainly appears in childhood, and the main onset is before 10-years of age.^45^ Topical corticosteroids, antihistamines, and systemic corticosteroids can be used to relieve symptomatic manifestations.^45^ Most photodermatoses can be treated with preventive UV phototherapy and/or PUVA. The goal of these treatments is to increase the patient’s tolerance to sunlight and prevent disease flare-ups.^46^ Although the exact mechanisms through which UVB and PUVA promote tolerance remain unclear, factors such as pigmentation and skin thickening may contribute significantly to the protective effect.^46^, ^47^ A retrospective study of 21 patients with actinic prurigo also found some success with phototherapy. One patient received BB-UVB therapy during the spring and early summer, with eight treatments totaling 750 seconds (approximately 15 MED). This patient reported positive outcomes in preventing new lesions. NB-UVB therapy was used in two other patients: one received 48 treatments with a cumulative dose of 42.1 J/cm^2^, and the other received 11 treatments with a cumulative dose of 2.7 J/cm^2^. PUVA therapy was also used in one patient, with both treatments proving beneficial. However, the positive effects of UV phototherapy were temporary, and the protective benefits diminished during the winter months unless therapy was continued.^48^ In a study involving five patients with actinic prurigo, PUVA treatment was administered twice weekly over 15-weeks. Before treatment, all patients exhibited heightened erythemal sensitivity to UVA and an abnormal enhancement of UVB erythema when treated with topical indomethacin. After undergoing PUVA therapy, all patients reported complete resolution of photosensitive symptoms, and the skin previously exposed to UVA showed normal erythemal responses.^49^

### Pityriasis lichenoides

**Fig. 4:**
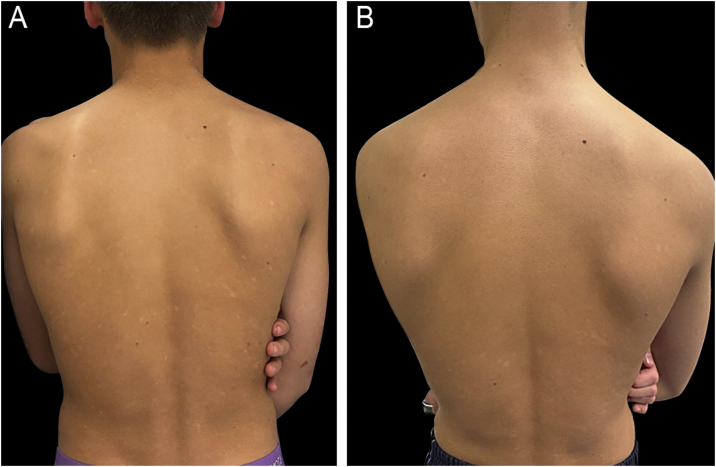
(A) Sixteen-year-old male patient diagnosed with chronic lichenoid pityriasis presenting with lichenified, scaly plaques on the lower back. (B) Same patient after 24 biweekly sessions of nb-UVB phototherapy, showing marked clinical improvement.

Pityriasis Lichenoides (PL) is a Papulosquamous inflammatory disease, with an acute form (PLEVA) that presents erythematous macules and papules that can present ulceration and heal with varioliform scarring, and a chronic form (PLC), that presents erythematous papules and plaques with a central scale with periodical relapses.^50^ The evidence for the use of phototherapy in treating PL comes from multiple small case series.^14^ NB-UVB is the most studied treatment modality for pediatric PL, in both its acute and chronic forms, with strong evidence supporting its efficacy (Fig. 4). A review of five patients (two with PLEVA and three with PLC) treated with NB-UVB showed complete remission in all five patients following therapy after an average of 21 sessions (range 13–40 sessions), corresponding to an average duration of therapy of 4-months (range 2–8 months). Each patient maintained remission at follow-up visits at 3- and 6-months.^51^ A systematic review of nine studies, involving 29 children treated with NB-UVB, found that 74% of patients achieved complete clearance, 13% had partial clearance, and 13% showed no improvement. On average, patients required 19 sessions to achieve a response.^6^ It is important to consider that phototherapy does not modify the course of the disease or its tendency to relapse, and there is no established consensus regarding its position in the PL therapeutic ladder. However, the use of NB-UVB is effective and well-tolerated and can be considered a first-line treatment for diffuse types or recurrent PL.^50^, ^52^

### Alopecia areata

Alopecia Areata (AA) is an autoimmune disease that is characterized by nonscarring hair loss usually in patches that can affect any part of the body.^53^ Its prevalence is approximately 2%, and in childhood, it has a prevalence of 0.11% and the peak is at 6‒9 years.^54^ The first line of treatment corresponds to the topical corticoids, followed by contact immunotherapy.^55^ In a retrospective review, Ersoy-Evans and colleagues reported on 10 children, aged 1 4 to 16, with varying severities of alopecia AA who were treated with PUVA. Of these patients, only 2 (30%) experienced complete hair regrowth.^20^ Similarly, another study assessing the use of NB-UVB for treating six children with AA found poor response rates, with 83% of patients reporting no improvement.^56^ Based on these findings and the lack of strong evidence supporting its efficacy, phototherapy is not currently recommended as a standard treatment for AA in the pediatric population. In contrast, some studies have shown the 308-nm excimer laser to be an effective treatment option for pediatric AA. In a study involving 9 children with AA, those treated with the excimer laser twice weekly for 12-weeks reported hair regrowth in 41.5% of the treated areas of the scalp, while no growth was observed in the untreated control areas.^57^ Additionally, a recent case report observed significant hair regrowth in a 5-year-old patient with extensive, refractory AA, treated with khellin and excimer laser.^58^

### Morphea/Localized scleroderma

Morphea corresponds to a rare autoimmune and inflammatory disease that affects the skin and subcutaneous tissue. It has a wide spectrum of clinical presentations, but it is usually characterized by the presence of erythematous plaques that progress with inflammation and then sclerosis and depigmentation of the affected zone.^59^ It´s more prevalent in Caucasians, and the mean age is between 7‒11 years.^60^ In most patients, the topical treatment can be enough, but in more widespread superficial disease, phototherapy (UVA1, UVA, PUVA, or UVB) and systemic treatment with MTX or MMF is indicated.^60^, ^61^ The current expert recommendation is the use of UVA1 or NB-UVB, preferably for children over 12-years of age with superficial, non-progressive morphea that does not involve joints or occur in “non-cosmetically sensitive areas”,^62^ and the suggested dose of UVA1 is 60 J/cm^2^ to a cumulative dose of 1460 J/cm^2^.^60^ Most of the available data supporting this approach comes from studies conducted on adults.^63^ In a prospective study involving 13 patients, including those in the pediatric age group, treatments with systemic PUVA, topical PUVA, and NB-UVB were evaluated, and all patients responded effectively to the treatment, regardless of the modality chosen, with improvements evident both clinically and through ultrasound examination.^64^

### Mastocytosis

Mastocytosis corresponds to a rare disorder characterized by the presence of abnormal accumulation and proliferation of mast cells in different tissues, and the most common is the skin, called “Cutaneous Mastocytosis” (CM). It is caused by mutations in the KIT gene.^65^ In pediatric patients, the more frequent form of presentation is mastocytosis limited to the skin (CM) and is considered a myeloproliferative clonal disease with a benign course, favorable outcome, and a tendency to spontaneously resolve before puberty.^66^ In a retrospective study of 20 patients with CM, 10 patients were treated with NB-UVB, and 10 with PUVA; both groups showed a statistically significant reduction in pruritus, along with a decrease in serum tryptase levels.^65^ UVB light has been shown to have an inhibitory effect on mast cell degranulation, likely by causing noncytotoxic damage to membrane phospholipid metabolism.^67^ Currently, there are no standard treatment guidelines for Systemic Mastocytosis (SM) in children. The primary treatment aim is to limit the release of mast cell mediators by avoiding potential triggering factors. The mainstay of systemic therapy consists of second-generation H1 and H2 antihistamines, which help reduce flushing, pruritus, and control wheal formation ^68^ and phototherapy (NB-UVB) can be considered a second-line therapy for refractory cases.^69^

### Langerhans cell histiocytosis

Phototherapy, specifically NB UVB, has also been explored as a therapeutic option for Langerhans Cell Histiocytosis (LCH) with cutaneous manifestations. However, only isolated cases are reported in the literature. Ness MJ et al. reported the use of NB-UVB in a pediatric patient with cutaneous LCH lesions, showing a favorable response with minimal adverse effects.^70^ Another report documented the successful treatment of a 10-month-old girl with cutaneous LCH using NB-UVB. Initially, the patient presented with an erythematous plaque on the chest, with the diagnosis confirmed after a biopsy. Since the lesion did not respond to triamcinolone injections, NB-UVB was chosen, resulting in complete resolution after 12-sessions with no adverse effects. These cases support the efficacy and safety of NB-UVB for managing cutaneous LCH lesions in children, avoiding the use of systemic treatments with potential side effects.^71^

### Cutaneous graft-versus-host disease

Cutaneous Graft-Versus-Host Disease (GVHD) is a common complication in patients with allogeneic bone marrow transplantation and can affect multiple organs, making the skin the most frequently involved.^72^ Typically, acute GVHD presents as a cutaneous rash, with erythematous, papular, or macular pruritic eruption.^73^ There are multiple treatment options, such as topical therapy with corticoids or calcineurin inhibitors, systemic therapy, and physical therapies (phototherapy or extracorporeal photopheresis).^74^ In a study of 10 pediatric patients with refractory response to first-line immunosuppressive treatment who were treated with NB-UVB achieved a complete remission was achieved in 80% of the patients.^75^ In a recent systematic revision, the authors evaluated a total of 28 studies and 1304 patients, were patients treated with PUVA the response rate was 89.9%, with a mean of 33.2 treatments, patients treated with NB-UVB response of 94%, with a mean number of 26 treatments, patients treated with UVA1 presents a respond of 89.3% with a mean of 26.2 treatments, but with higher adverse events reported.^72^ PUVA and UVA1 appear to be better for the treatment of sclerotic skin manifestations.^74^

### Prurigo nodularis

Prurigo nodularis is a chronic skin disease that is characterized by the presence of multiple itchy nodules on the skin. For this disease, the authors found only one case of a patient with nodular prurigo treated with 25 sessions of NB UVB phototherapy, achieving a total cumulative dose of 18.0 J/cm^2^. After treatment, significant improvement was observed, with skin that was virtually free of lesions according to the PGAS scale. However, the patient was lost to follow-up at 12-months.^1^

### Other conditions

Phototherapy has also been used to treat other conditions in pediatric patients, such as urticaria pigmentosa, hydroa vacciniforme, and pruritic dermatoses.^4^ Additionally, phototherapy may be used as photo-prophylaxis for light-sensitive cutaneous eruptions, such as erythropoietic protoporphyria and polymorphic light eruption.^76^

### Practical considerations

According to the authors’ experience and the publications on the subject, for proper selection of candidates, it is essential to conduct a detailed medical history and thorough physical examination and consider the presence of absolute contraindications. Once these have been ruled out, the most appropriate phototherapy modality and optimal treatment protocol should then be selected.^13^ It is also critical to obtain written informed consent from the parents or guardians before initiating treatment, ensuring they understand the benefits, risks, alternatives, and expected course of phototherapy.

Dosing and frequency protocols for phototherapy in children can vary, as there are no established guidelines for treatment parameters. Typically, the treatment plan begins with calculating the Minimal Erythema Dose (MED), with the starting dose set at 70% of the MED or lower. Subsequent sessions usually increase by 20% per treatment. Some studies, however, suggest a more gradual escalation of 10% per session to minimize acute side effects such as erythema and burning and to improve overall tolerability.^1^ It should be noted that the initial treatment dose can also be calculated based on the patient's skin type, with progressive increases. Adjustments may be required if there are long intervals between sessions. In the case of PUVA therapy, baseline, and follow-up laboratory tests such as liver function tests, renal profile, and complete blood count, among others, should be performed due to the systemic administration of psoralens. Additionally, psoralen formulations should be carefully selected for pediatric patients, with preference for low-dose or liquid preparations to ensure safe and accurate dosing.^77^

A retrospective study involving 98 pediatric patients with a mean age of 10.5 years and 122 adults receiving phototherapy showed no statistically significant differences in dosage, duration, or number of sessions between the two groups when treated with NB-UVB therapy or PUVA. A complete response was achieved in 35% of pediatric patients, and no differences were found between the pediatric and adult groups. This study concluded that NB-UVB therapy and PUVA are safe and effective for children and can follow the same treatment protocols used in adults. The most common conditions treated were psoriasis (48%), vitiligo (17%), and atopic dermatitis (16%).^78^

Administering phototherapy to children presents several challenges. One of the main difficulties is ensuring compliance with protective eyewear, especially in younger children, and ensuring that children remain still during treatment, which is crucial for targeting the affected areas and minimizing side effects.^4^ Therefore, school-age children are often considered the most reasonable starting point for UVB therapy. It is also important to assess the child's behavioral development, including their ability to remain still and manage separation anxiety.^14^ It is essential to implement comprehensive photoprotection measures both during and after phototherapy sessions, as the skin becomes more sensitive to UV radiation following treatment. Physical photoprotection, such as the use of protective clothing, including balaclavas or garments covering uninvolved areas, is particularly important during phototherapy to shield unaffected skin from unnecessary exposure.^4^, ^7^, ^14^ In parallel, rigorous chemical photoprotection through the daily application of broad-spectrum sunscreens is strongly recommended outside of treatment sessions to reduce the risk of cumulative UV-induced damage. In this context, the importance of patient and family education and the value of obtaining written informed consent before initiating treatment are crucial.

Another challenge is the frequency of treatment sessions, which usually occur 2 to 3 times per week. This can be difficult for both the child and the parent, especially for school-aged children who may miss school time due to frequent treatments.^4^ Adherence to treatment is one of the principal challenges in phototherapy, but studies suggest that in the pediatric population, adherence is higher than in adults. In a recent study, the main cause of incomplete treatment the school incompatibility.^78^ In selected cases, home phototherapy may be considered to improve adherence and reduce logistical burdens. However, this approach requires rigorous training for caregivers, appropriate safety measures, regular medical supervision, and a clear understanding of the risks, such as burns or overexposure.^30^

To address these challenges, it is recommended that children and their parents familiarize themselves with the treatment unit. They should be allowed to enter and exit the unit as needed. During treatment, parents may stay nearby, with the option of keeping the door slightly ajar or standing outside to comfort the child. Over time, the goal is to transition to unaccompanied sessions.^14^

### Adverse effects and safety considerations

The principal acute adverse effects of phototherapy, depending on the modality, include erythema, pruritus, xerosis, and tanning, and the long-term effects include the risk of photocarcinogenesis and premature skin aging.

For UVB and UVA phototherapy, short-term side effects include erythema (appears in the first 24-hs), blistering, xerosis, pruritus, photosensitive eruptions, and recurrent herpes simplex virus infections. Also, the risk of phototoxic reactions, such as tetracyclines, fluoroquinolones, voriconazole, azathioprine, amiodarone, and others should be considered. Anxiety may also arise in some patients and should be discussed before treatment. However, these side effects can be controlled with the education of the patient and correct selection of modality, dosing and dose adjustment, protection of the unevolved areas, monitoring of cumulative UV doses, and full-body skin examination.^7^ There is no measurable evidence of increases in photocarcinogenesis in patients treated with NB-UVB and UVA1.^2^, ^79^, ^80^

In clinical practice, the decision to initiate phototherapy must be preceded by a careful evaluation of contraindications and precautionary conditions. These include absolute contraindications (e.g., photosensitivity syndromes, history of melanoma), relative contraindications (e.g., pregnancy, immunosuppression, pediatric age), and specific precautions (e.g., use of photosensitizing drugs, previous radiation exposure, ocular protection) that vary slightly depending on the modality used (NB-UVB vs. PUVA), a detailed summary of these conditions are provided in Table 2. This stratification is essential for optimizing safety and minimizing risk, particularly in vulnerable populations.^13^, ^14^, ^81^

**Table 2:** Contraindications and considerations of the use of UVB and PUVA phototherapy.^4^, ^81^

Contraindication	UVB phototherapy	PUVA phototherapy
Absolute	• Genetic syndromes with photosensitivity or cancer risk (e.g., xeroderma pigmentosum, Bloom syndrome, Gorlin syndrome, Cockayne syndrome). • Photosensitive dermatoses (e.g., systemic lupus erythematosus, dermatomyositis). • Inability to follow or realize treatment protocol (e.g., severe claustrophobia, physical limitations).	• Genetic syndromes with photosensitivity or cancer risk (e.g., xeroderma pigmentosum, Bloom syndrome, Gorlin syndrome, Cockayne syndrome). • Photosensitive dermatoses (e.g., systemic lupus erythematosus, dermatomyositis). • Pregnancy and breastfeeding (oral PUVA). • Severe Hepatic or renal impairment (oral PUVA). • Hypersensitivity or intolerance to psoralen. • Untreated cataracts.
Relative	• History of melanoma or non-melanoma skin cancer. • Previous exposure to arsenic or ionizing radiation. • Use of photosensitizing agents (e.g., tetracyclines, retinoids, NSAIDs). • Photodermatoses (e.g., chronic actinic dermatitis, polymorphous light eruption, solar urticaria). • Immunosuppression (e.g., transplant recipients, systemic immunosuppressants). • Cataracts without adequate eye protection. • Significant systemic disease (e.g., liver/kidney failure).	• History of melanoma or non-melanoma skin cancer. • Current premalignant skin lesions. • Prior arsenic or radiation exposure. • Bullous pemphigoid/Pemphigus. • Immunosuppression. • Hepatic or renal impairment. • Allergy or intolerance to psoralens. • Uncontrolled epilepsy (risk of light-induced seizures).
Precautions	• Pregnancy (requires folate supplementation and UV dose control; and extra facial protection to prevent melasma). • Multiple atypical nevi / FAMMM (familial atypical multiple mole melanoma) syndrome. • Use of photosensitizing medications or herbs. • History of excessive UV exposure or photodamage. • Outdoor occupations (additional cumulative UV exposure). • Children under 16-years (use cautiously in ages 6–16).	• Ophthalmologic evaluation. • Over 200 lifetime PUVA sessions (increased risk of squamous cell carcinoma). • Genital Male protections (increased cancer risk). • Patients with cognitive or adherence difficulties. • History of excessive UV exposure or photodamage • Outdoor occupations (additional cumulative UV exposure). • Children under 10 (prefer NB-UVB in pediatric cases).

NSAIDs, Non-Steroidal Anti-Inflammatory Drugs.

Some studies have reported safe cumulative doses of up to 1985 mJ/cm^2^ in dark-skinned populations without observing cutaneous malignancy during long-term follow-up. The AAD guidelines recommend maximum doses per session that vary according to phototype, reaching up to 5000 mJ/cm^2^ for phototypes V–VI.^82^ However, they do not establish an absolute limit for cumulative dose or number of sessions. In clinical practice, between 50 and 200 sessions are typically performed, and there are reports of patients treated with over 500 sessions without a significant increase in skin cancer risk.^34^, ^83^ On the other hand, it is known that the risk of melanoma increases significantly after more than 200 PUVA sessions, particularly after 15-years of follow-up.^82^ Regarding cumulative dose, studies in dark-skinned populations have reported up to 2085 J/cm^2^ of UVA without observing malignancy. However, in fair-skinned populations, it is recommended to limit cumulative exposure and the total number of sessions, ideally not exceeding 150–200 sessions over a lifetime.^84^ For UVA1, protocols vary, but at least 8 sessions with medium-to-high doses (up to 40 J/cm^2^ per session) are generally required for a clinical response. No absolute limit for cumulative dose or maximum number of sessions has been established in the reviewed literature.^85^

For PUVA, oral psoralens can cause nausea and vomiting.^86^ Short-term side effects of PUVA include erythema (which appears in 48–72 hours), swelling, and blister formation. Additionally, PUVA therapy can induce cataracts, and because the ocular lens is more permeable at a younger age, oral PUVA is relatively contraindicated in children younger than 12-years old.^87^ Long-term effects of PUVA include photoaging, pigmentary changes, rhytides, xerosis, actinic damage, and PUVA lentiginosis (freckling).^14^, ^81^ Evidence has found that PUVA is associated with a significantly increased risk of developing Squamous Cell Carcinoma (SSC) and Actinic Keratoses (AK) principally, but also, in lower incidence, in melanoma and Basal Cell Carcinoma (BCC).^8^, ^80^

Absolute contraindications for phototherapy include photosensitive and skin cancer-predisposing disorders, such as xeroderma pigmentosum, systemic lupus erythematosus, and Gorlin syndrome.^13^ A family history of skin cancer should also be considered, although it is not necessarily a contraindication.^14^

A monocentric retrospective study of 90 children under the age of 16 who received a total of 790 phototherapy sessions (averaging three sessions per week) found that phototherapy was generally well tolerated. Among these patients, 52% were treated for psoriasis, with other indications including vitiligo (19%), atopic dermatitis (11%), pruritus/prurigo (9%), and alopecia areata (9%). Mild erythema occurred in 15% of the patients, and 32% of patients discontinued treatment, primarily due to difficulty balancing treatment schedules with parental employment or children's school hours.^3^ A retrospective review of 100 children under 18 who received NB-UVB, BB-UVB, or PUVA photochemotherapy also found that treatments were well tolerated. Grade 2 erythema or higher occurred in 46% of children, but more severe reactions (Grade 3 and 4) were infrequent, and only three children stopped treatment due to burning.^10^

The primary long-term risk of phototherapy in children is the potential development of cutaneous malignancy due to repeated UV exposure. However, this potential risk must be weighed against the benefits of treatment. A retrospective cohort study involving 3,506 patients, 16 to 100 years, with a median age of 50-years at follow-up, treated with BB-UVB, NB-UVB, and/or combined UVA, with a mean follow-up of 7.3-years, assessed the incidence of skin cancer. Most patients had psoriasis (60.9%) or eczema (26.4%), and the median number of treatments was 43 (ranging from 1 to 3,598). Overall, 170 skin cancers were reported, including 17 melanomas, 33 SCC, and 120 BCC. There was no significant difference in the incidence of these skin cancers compared to the general population, and no cumulative dose-response correlation between UVB exposure and skin cancer was observed.^88^ However, this potential risk warrants special consideration in children due to their longer life expectancy and the possibility of increased carcinogenic risks that may become evident later in life.^4^

### Phototherapy in the era of biologics

Phototherapy remains one of the most evidence-based and safest treatment modalities in dermatology for managing a range of skin diseases. Phototherapy is one of the most evidence-based and safe treatments in dermatology for managing various skin diseases. Its most common indications include psoriasis, vitiligo, and atopic dermatitis, among others. However, the emergence of new therapies, such as biological drugs, has raised questions about the continued role of phototherapy within the treatment landscape for these conditions. For example, in vitiligo, the combination of topical JAK inhibitors, such as ruxolitinib, with NB-UVB phototherapy may exert a synergistic effect on repigmentation. However, robust evidence is currently limited to adult populations, and further data are needed in pediatric patients.^89^

A comparative study that evaluated the clinical response of patients with psoriasis treated with oral PUVA versus biologic treatments showed that oral PUVA was comparable to the one treated with infliximab and superior to etanercept, efalizumab, adalimumab, and Ustekinumab.^90^ A recent patient-reported study of patients with psoriasis treated with NB-UVB versus adalimumab versus placebo showed that both therapies improve skin-related quality of life and overall health-related quality of life after 12 weeks of treatment.^91^

The cost of a treatment is also an important factor to consider; an analysis made in 2010 estimated that biologic agents, such as adalimumab, cost at least twice that of NB-UVB and PUVA combined.^7^, ^92^ Despite significant advancements in the management of dermatological diseases, phototherapy remains the treatment of choice for patients with underlying conditions that preclude the use of systemic therapies, a history of toxicity, drug interactions, refractoriness to systemic treatments, or economic considerations.^93^

## Conclusion

Phototherapy is an effective and well-tolerated treatment option for pediatric patients with various skin conditions, including psoriasis, atopic dermatitis, vitiligo, and others. The decision to initiate phototherapy in pediatric patients should be made on a case-by-case basis, considering factors such as the severity and extent of the condition, previous treatment responses, the child's age and behavior, as well as the family's ability to consistently adhere to the treatment regimen. NB-UVB is the widely used and preferred modality due to its efficacy and lower risk, is well tolerated in children, but there is a clear need for studies reviewing its long-term side effects. It is important to note that treatment protocols in this population often lack standardization and are frequently extrapolated from adult experiences, underscoring the urgent need to develop pediatric-specific guidelines. While current evidence supports the use of phototherapy in children, long-term studies are still needed to establish optimal dosing regimens, better define safety over time, and evaluate its true impact on quality of life. In this regard, the implementation of innovative strategies, such as home-based phototherapy, could represent a significant advance in accessibility and treatment adherence.

## ORCID ID

Eine Benavides: 0000-0002-2064-2647

Catalina Retamal: 0009-0008-4775-3911

Fernando Valenzuela: 0000-0003-1032-9347

## Financial support

This research has not received specific support from public sector agencies, the commercial sector, or non-profit entities.

## Authors’ contributions

Eine Benavides: Critical literature review; Manuscript critical review; Preparation and writing of the manuscript.

Dan Hartmann: Approval of the final version of the manuscript; Critical literature review; Manuscript critical review; Preparation and writing of the manuscript.

Catalina Retamal: Critical literature review; Manuscript critical review; Preparation and writing of the manuscript.

Fernando Valenzuela: Approval of the final version of the manuscript; Critical literature review; Intellectual participation in propaedeutic and/or therapeutic management of studied case; Manuscript critical review; Preparation and writing of the manuscript.

## Research data availability

The entire dataset supporting the results of this study was published in this article.

## Conflicts of interest

The authors declare that they have no conflicts of interest in this publication.
